# Catecholamine Surges Cause Cardiomyocyte Necroptosis *via* a RIPK1–RIPK3-Dependent Pathway in Mice

**DOI:** 10.3389/fcvm.2021.740839

**Published:** 2021-09-16

**Authors:** Penglong Wu, Mingqi Cai, Jinbao Liu, Xuejun Wang

**Affiliations:** ^1^Division of Basic Biomedical Sciences, University of South Dakota Sanford School of Medicine, Vermillion, SD, United States; ^2^Guangzhou Municipal and Guangdong Provincial Key Laboratory of Protein Modification and Degradation, State Key Laboratory of Respiratory Disease, School of Basic Medical Sciences, Affiliated Cancer Hospital of Guangzhou Medical University, Guangzhou, China

**Keywords:** necroptosis, RIPK1, RIPK3, isoproterenol, cardiomyocyte, mice, catecholamine surge, COVID-19

## Abstract

**Background:** Catecholamine surges and resultant excessive β-adrenergic stimulation occur in a broad spectrum of diseases. Excessive β-adrenergic stimulation causes cardiomyocyte necrosis, but the underlying mechanism remains obscure. Necroptosis, a major form of regulated necrosis mediated by RIPK3-centered pathways, is implicated in heart failure; however, it remains unknown whether excessive β-adrenergic stimulation-induced cardiac injury involves necroptosis. Hence, we conducted the present study to address these critical gaps.

**Methods and Results:** Two consecutive daily injections of isoproterenol (ISO; 85 mg/kg, s.c.) or saline were administered to adult mixed-sex mice. At 24 h after the second ISO injection, cardiac area with Evans blue dye (EBD) uptake and myocardial protein levels of CD45, RIPK1, Ser166-phosphorylated RIPK1, RIPK3, and Ser345-phosphorylated MLKL (p-MLKL) were significantly greater, while Ser321-phosphorylated RIPK1 was significantly lower, in the ISO-treated than in saline-treated wild-type (WT) mice. The ISO-induced increase of EBD uptake was markedly less in *RIPK3*^−/−^ mice compared with WT mice (*p* = 0.016). Pretreatment with the RIPK1-selective inhibitor necrostatin-1 diminished ISO-induced increases in RIPK3 and p-MLKL in WT mice and significantly attenuated ISO-induced increases of EBD uptake in WT but not RIPK3^−/−^ mice.

**Conclusions:** A large proportion of cardiomyocyte necrosis induced by excessive β-adrenergic stimulation belongs to necroptosis and is mediated by a RIPK1–RIPK3-dependent pathway, identifying RIPK1 and RIPK3 as potential therapeutic targets for catecholamine surges.

## Introduction

In response to physical (e.g., cardiac failure and stroke) or emotional stressors, the sympathetic nervous system and the hypothalamic–pituitary–adrenal axis become hyperactive and give rise to catecholamine surges and cardiac injury (1–3). Catecholamine surges can also occur in some of the less common clinical conditions. For example, in patients with pheochromocytoma or paraganglioma, the chromaffin cells in the tumor can secrete large amounts of adrenalin or norepinephrine into the circulation, causing hypertension, cardiac injury, and damages to other organ systems (4–6); patients with Irukandji syndrome that is caused by the sting of a type of jellyfish show symptoms of catecholamine surges (7). Catecholamine surges can also result from clinical treatment (8). The catecholamine surge condition most relevant to the current COVID-19 pandemic is arguably Takotsubo syndrome, which is often triggered by psychological and physical stressors. Takotsubo cardiomyopathy, also known as broken heart syndrome or stress cardiomyopathy, can occur in COVID-19 patients (9–11). Catecholamine surges have been proposed as an important pathogenic factor for Takotsubo cardiomyopathy, including the one associated with COVID-19 (12). Interestingly, Jabri et al. reported that the incidence of Takotsubo cardiomyopathy diagnosed in patients with acute coronary syndrome (ACS) during the COVID-19 pandemic (between March 1 and April 30, 2020) in the northeast Ohio area rose to 7.8%, compared with multiple control groups of patients with ACS presenting before the pandemic across four distinct timelines, which had showed incidences ranging from 1.5 to 1.8% (13), suggesting that psychological stress associated with the COVID-19 pandemic may increase the incidence of Takotsubo cardiomyopathy even in non-COVID-19 patients. Therefore, a better understanding of the pathogenic mechanism of catecholamine surges will help advance the pathophysiology of a broad spectrum of human diseases.

Cardiac injury has long been observed in diseases associated with catecholamine surges. As highlighted by Watkins in a review article published in 1957 (14), myocardial fibrosis and inflammatory responses were observed in patients suffering from pheochromocytoma, a rare but treacherous catecholamine-producing tumor. More than a century ago, intravenous injections of adrenalin were shown to induce myocarditis and the morphological features of cardiomyocyte degeneration and necrosis in rabbits (15). In late 1950s, Rona et al. reported an infarct-like myocardial lesion produced by subcutaneous injections of a synthetic catecholamine isoproterenol (ISO) in rats (16, 17), which recapitulates many aspects of myocardial lesions previously described for patients with pheochromocytoma or paraganglioma (5, 18). Subsequently, the induction of cardiomyocyte necrosis in rats or mice by two consecutive daily injections of high dosage ISO was used by many as a non-invasive method to model acute myocardial injury or even model myocardial infarction (MI) (19–21). Mechanisms by which ISO induces cardiac necrosis remain ill-defined, although reported studies have suggested numerous theories, such as coronary insufficiency, oxidative stress, altered metabolism, and ionic imbalance (21).

During apoptosis or non-lytic cell death, cell membrane permeability is not increased so that the cellular content of an apoptotic cell *in vivo* does not leak into the extracellular space; even the apoptotic bodies derived from disintegration of the apoptotic cell are sequestered by the membrane before they are removed by phagocytes (22). During necrotic or lytic cell death (23), however, the dying cell loses its cell membrane integrity or the control of membrane permeability, allowing free movement of water and other high-molecular-weight molecules across the cell membrane and rendering the cell to swell and ultimately burst. The leak of intracellular components into the interstitial space causes inflammation. Hence, different from apoptosis, necrosis is always accompanied by inflammatory responses (22). Recent advances in our understanding of cell death have classified necrosis into two major categories: accidental/passive necrosis and regulated necrosis. The former results from direct physical or chemical insults to the cell that directly destroy the cell membrane and break the cell; so it happens instantly and is not voluntarily controllable by the demising cell. By contrast, the regulated necrosis is triggered by biochemical changes inside or outside of the cell and takes a pathway that is intrinsically controllable by the affected cell (24). Under the category of regulated necrosis, several types have emerged, such as necroptosis, ferroptosis, pyroptosis, and mitochondrial permeability transition (MPT) pore-dependent necrosis (24). Since these pathways to regulated necrosis could potentially be intervened to prevent the necrosis from occurring, deciphering the nature of, and delineating the molecular pathways to, the necrosis in diseased organs can pave new avenues to devising new therapeutic strategies for the disease. Hence, we sought to define the nature of cardiomyocyte necrosis induced by catecholamine surges in the present study.

Upon TNFα receptor 1 (TNFR1) stimulation, most cells undergo apoptosis, but the cells with caspase 8 deficiency or caspase inhibition undergo necrosis, instead. The latter is termed necroptosis (25). The canonical pathway mediating TNFα-induced necroptosis requires the kinase activity of receptor-interacting protein kinase 1 (RIPK1) (26). RIPK1 binds and phosphorylates RIPK3, and the phosphorylated RIPK3 further phosphorylates a pseudo kinase known as mixed lineage kinase domain-like protein (MLKL) (27); then the phosphorylated MLKL (p-MLKL) is believed to translocate to the plasma membrane and oligomerize to form pores on the cell membrane (28), thereby increasing membrane permeability and causing the cell to swell and ultimately burst (29). Some more recent studies suggest that necrosomes formed by RIPK3 and p-MLKL oligomers are indispensable to necroptosis and that not all necroptosis requires RIPK1, but RIPK1 kinase activity is required for TNFα stimulation to induce necroptosis (22).

Cardiomyocyte death including apoptosis and various forms of regulated necrosis contributes to cardiac pathogenesis (22). The myocardium from humans with end-stage heart failure resulting from MI or dilated cardiomyopathy displayed elevation of necroptotic biochemical markers, indicative of an involvement of necroptosis in heart failure (30). Heart failure patients harboring a genetic variant in the promoter region of *RIPK3* gene that increases *RIPK3* gene expression tend to exhibit poorer prognosis than those who do not carry such a variant (31). Experimental studies have demonstrated an important pathogenic role for necroptosis in common pathological processes such as post-MI remodeling (32), myocardial ischemia/reperfusion (I/R) injury, cardiotoxicity of doxorubicin treatment (33, 34), and paraquat-induced cardiac contractile dysfunction (35). Therefore, a better understanding of the cellular and molecular mechanisms that govern cardiomyocyte necroptosis or link pathological stress to cardiomyocyte necroptosis is expected to unveil new therapeutic targets to prevent or more effectively treat heart failure.

Using primarily the ISO-induced rodent models of cardiac injury since late 1950s, researchers have attributed many factors to the cardiac injury induced by catecholamine surges and excessive β-adrenergic stimulation. Elevated oxidative stress is a well-known damaging factor to the cell. Myocardial oxidative stress is drastically increased either by the myocardial I/R as a result of coronary spasm and subsequent release or by the metabolism of catecholamines, as some of the catecholamine metabolites are strong oxidants (36). Calcium overload and myofibril over-contraction, which can be a direct result of excessive β-adrenergic stimulation from catecholamines and a secondary consequence of I/R injury, may contribute to cardiac dysfunction and injury (36). Additionally, β-adrenergic stimulation appears to be able to trigger inflammatory responses by upregulating the expression and release of inflammatory cytokines (37); and many compounds with an anti-inflammatory property can protect against cardiac injury induced by catecholamine surges (21), suggesting that the secondary injury from inflammation may also play a role in the cardiac injury by catecholamine surges. Necrotic cardiomyocyte death is the most prominent pathological feature of cardiac injury induced by catecholamine surges (19). However, the nature of cardiomyocyte necrosis induced by catecholamine surges or by excessive β-adrenergic stimulation remains undefined. Hence, we conducted the present study to determine if the necroptotic pathway plays a role in mediating cardiomyocyte necrosis induced by ISO. Our findings provide compelling evidence for the first time that a large proportion of cardiomyocyte necrosis induced by catecholamine surges belongs to necroptosis and is mediated by the RIPK1–RIPK3–MLKL pathway, and we have further demonstrated that targeting RIPK1 or RIPK3 can significantly attenuate ISO-induced necrosis, providing strong evidence for targeting RIPK1 and RIPK3 to protect the heart against injury from catecholamine surges or excessive β-adrenergic stimulation.

## Methods and Materials

### Animal Models

The creation of RIPK3 global knockout (*RIPK3*^−/−^) mice was previously described (38). The *RIPK3*^−/−^ mice used in this study had undergone more than nine generations of back-crossing into the C57BL/6J inbred background. All mice used here are in the C57BL/6J inbred background. A recent report has documented that there is no sex difference in the induction of cardiac dysfunction by an ISO treatment regime in mice (39). Hence, young adult age- and sex-matched wild-type (WT) or *RIPK3*^−/−^ mice were randomly divided into two groups and subjected to two consecutive subcutaneous injections of ISO (#I6504, Sigma-Aldrich, St. Louis, MO, USA; 85 mg/kg) or an equivalent amount of vehicle control [saline (SAL)], with an interval of 24 h between the two injections. The rationale for choosing this ISO dosage and treatment regime is that it has been extensively used in prior studies by others to induce MI-like cardiac injury reproducibly, although two consecutive daily injections of ISO at a dose as low as 0.33 mg/kg could induce cardiomyocyte necrosis in rats (21). To test the effect of RIPK1-selective inhibition, necrostatin-1 (NEC-1; BML-AP309-0020, Enzo Life Sciences, Inc., Farmingdale, NY, USA; 4 mg/kg, i.p.) was administered 10 min before each injection of ISO (or saline). For further protein biochemistry and histopathology analyses, the ventricular myocardium was collected 24 h after the second ISO injection. The animal care and use protocol for this study was approved by the Institutional Animal Care and Use Committee of the University of South Dakota.

### Evans Blue Dye Uptake Assays

The *in vivo* Evans blue dye (EBD) uptake assay was performed to detect cardiomyocyte necrosis as we reported (40, 41). After it is absorbed into the circulatory system, EBD is bound by albumin. Therefore, in this assay, EBD does not enter cells with an intact plasma membrane (40). EBD (#314-13-6, Sigma-Aldrich) was dissolved in saline (10 mg/ml). Mice were intraperitoneally injected with EBD (100 μg/g body weight) 18 h before tissue collection. To flush out EBD in the vasculature and the interstitial space, the heart was retrogradely perfused *via* the abdominal aorta (distal end ligated) first with phosphate-buffered saline (PBS; pH 7.4) for 5 min; and for *in situ* fixation, the PBS was then replaced with 4% paraformaldehyde. The fixed ventricular myocardium was equilibrated with 50% sucrose for 4 h before being embedded in O.C.T. (Sakura Finetek USA, Inc., Torrance, CA, USA); the tissue block was then frozen in liquid nitrogen and stored in a −80°C freezer until being sectioned. Cryosections (7 μm) were collected, washed with PBS, and counterstained with Alexa Fluor™ 488 Phalloidin and DAPI (4′,6-diamidino-2-phenylindole) to reveal F-actin (green) and nuclei (blue), respectively. Stained sections were imaged using a Leica TCS SP8 STED 3X White Light Laser and Super-Resolution Confocal Imaging System (Leica Microsystem, Buffalo Grove, IL, USA). Cells that have taken up EBD show red auto-fluorescence and are readily identifiable. An image of the whole section was generated with the tiling function of the built-in imaging software. The percentage of the EBD positive area (red) over the total myocardial area (green and red) was measured by ImagePro Plus 6.0 software from representative sections from each mouse.

### Co-Immunoprecipitation

Co-immunoprecipitation (Co-IP) was done with the Pierce Co-IP Kit (Catalog 26149, Thermo Fisher Scientific, Waltham, MA, USA), which provides covalent antibody immobilization so that potential interference of the immunoprecipitation antibodies is completely avoided. The mouse monoclonal antibody against RIPK1 (#610459; BD Biosciences, San Jose, CA, USA) was used for IP and immunoblot of RIPK1; immunoblot for RIPK3 used the rabbit polyclonal anti-RIPK3 (ADI-905-242-100; Enzo).

### Protein Extraction and Western Blotting Analyses

The extraction of total proteins from ventricular myocardial samples was done using 1 × loading buffer containing 41 mM of Tris-HCl, 1.2% sodium dodecyl sulfate (SDS), and 8% glycerol. A protease inhibitor cocktail (#P-1540; AG Scientific, San Diego, CA, USA) was added to the extraction buffer to inhibit protein degradation. Protein concentration was determined using bicinchoninic acid reagents (#23225; Thermo Fisher Scientific, Waltham, MA, USA). Equal amounts of proteins loaded to different lanes were fractionated *via* 8–14% SDS–polyacrylamide gel electrophoresis (SDS-PAGE), and the separated proteins were transferred onto a polyvinylidene difluoride (PVDF) membrane using a trans-blot apparatus (Bio-Rad, Hercules, CA, USA). The PVDF membranes were then sequentially subjected to blocking, incubation with the primary antibodies against the protein of interest, washing with the TBST (Tris-buffered saline with 0.1% Tween^®^ 20 detergent) buffer to remove unbound primary antibodies, incubation with horseradish peroxidase (HRP)-conjugated secondary antibodies (Santa Cruz Biotechnology, Dallas, TX, USA), and washing to remove unbound antibodies. The secondary antibodies bound to the PVDF membrane were then detected using enhanced chemiluminescence reagents (GE Healthcare, South Plainfield, NJ, USA); the chemiluminescence was digitally imaged and analyzed with the ChemiDoc™ MP imaging system and associated software (Bio-Rad, Hercules, CA, USA) as we previously reported (42). The stain-free total protein imaging technology was used as described to obtain the image from the gel and PVDF membrane to be used as loading controls (43).

### Statistical Methods

GraphPad Prism software (Version 8.4; GraphPad Software, San Diego, CA, USA) was used. All continuous variables are presented as scatter dot plots with mean ± SEM superimposed. All data were examined for normality with the Shapiro–Wilk test prior to application of parametric statistical tests. Tests used for statistical significance evaluations of each dataset are specified in the figure legends. The difference between the two groups was evaluated using two-tailed unpaired *t*-test with Welch's correction to address the potential issues associated with small sample size or, where technical repeats are involved, nested *t*-test. One-way ANOVA or nested one-way ANOVA or when appropriate, two-way ANOVA, followed by Tukey's multiple comparisons test was used to evaluate the difference among three or more groups. A *p*-value or, where applicable, adjusted *p*-value < 0.05 is considered statistically significant.

## Results

### Induction of Massive Cardiomyocyte Necrosis by High Doses of Isoproterenol

**Figure 1:**
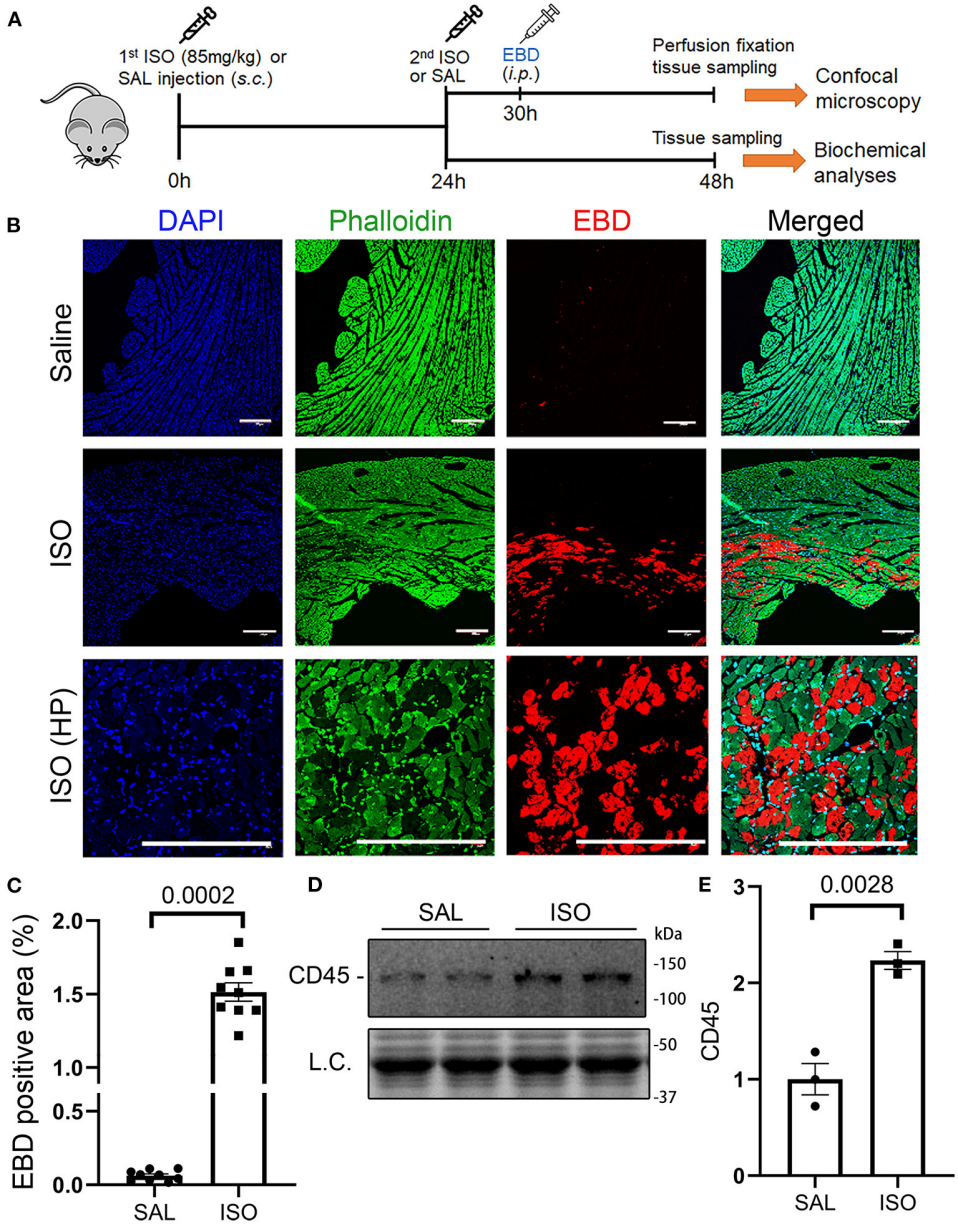
Isoproterenol (ISO) treatment induced cardiomyocyte necrosis in wild-type mice. **(A)** A schematic for the design of experiments presented in this figure and in Figures 2, 3. Adult mice of both sexes were subjected to two daily subcutaneous injections of ISO (85 mg/kg) or vehicle control [saline (SAL)] with an interval of 24 h. Myocardial tissue was collected at 24 h after the second dose of ISO. Evans blue dye (EBD) was injected 18 h before tissue collection. EBD in the vasculature and extracellular space was flushed out through retrograde perfusion with saline *via* the abdominal aorta immediately before tissue collection. A separate cohort without EBD injection and perfusion fixation was used for biochemical analyses. **(B,C)** *in vivo* EBD uptake assays. Cryosections were used for staining with Alexa 488-conjugated phalloidin and DAPI before imaging with a multicolor confocal microscope. Shown are confocal micrographs centered on an EBD positive area in the ISO-treated heart or the corresponding region of a saline-treated heart **(B)** and a graph summarizing the percentage EBD-positive area from three mice (two males and one female) per group **(C)**. Scale bar = 200 μm; mean ± SEM; three sections/mouse and three mice/group were included; nested *t*-test. HP, higher magnification. **(D,E)** Representative image **(D)** and pooled densitometry data **(E)** of Western blotting analyses for myocardial CD45. *N* = 3 mice (two males and one female) per group; the *p*-value shown in panel E is derived from two-sided unpaired *t*-test with Welch's correction. L.C. (loading control) used total protein images obtained with the stain-free total protein imaging technology.

The hallmark between necrosis and apoptosis is the loss of membrane integrity of the cell undergoing necrosis, whereas the cell membrane permeability does not increase during *in vivo* apoptosis. As a result, inflammatory responses are triggered by necrosis but not by apoptosis. Hence, we performed the EBD uptake assay to assess cardiomyocyte plasma membrane integrity in mice subjected to ISO or saline treatment. EBD administered *via* a peritoneal injection was not found in the cardiomyocyte compartment of saline treated mice, but a significant proportion of cardiomyocytes in the ISO treated mice contained EBD in their cytoplasm (Figures 1B,C; Supplementary Figure I), indicative of the loss of plasma membrane integrity in these cardiomyocytes. In response to necrosis, leukocyte infiltration ensues. Our Western blotting analyses showed that myocardial protein levels of CD45, a leukocyte marker, were markedly higher in ISO-treated mice than in the saline-treated group (Figures 1D,E, *p* = 0.003), which is further consistent with occurrence of necrosis in ISO-treated mouse hearts.

### Activation of the Myocardial RIPK1–RIPK3–MLKL Pathway in Isoproterenol-Treated Mice

**Figure 2:**
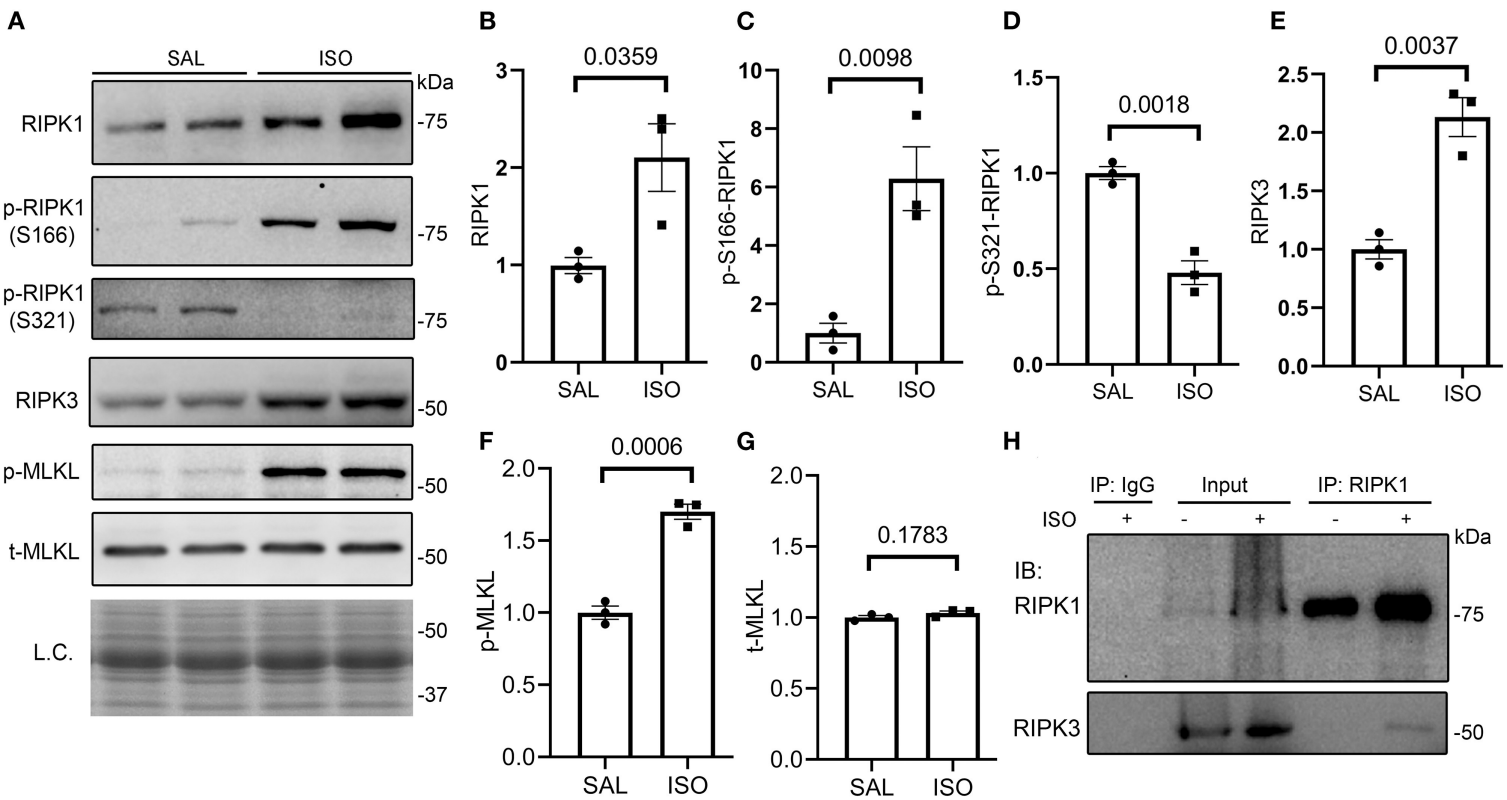
Myocardial RIPK1–RIPK3–MLKL pathway is activated in isoproterenol (ISO)-treated mice. Mice were treated with ISO or saline (SAL) as described in Figure 1. Ventricular myocardial samples were collected at 24 h after the second dose of ISO for further analyses, and total proteins were extracted for Western blotting analyses for the indicated proteins. **(A–G)** Representative images **(A)** and pooled densitometry data **(B–G)** of Western blotting analysis for the indicated proteins. p-S166-RIPK1, Ser166-phosphorylated RIPK1; p-S321-RIPK1, Ser321-phosphorylated RIPK1; p-MLKL, Ser345-phosphorylated MLKL; t-MLKL, total MLKL; L.C., loading control that used the stain-free total protein image; mean ± SEM; each dot represents a mouse; *n* = 3 mice (two males and one female) per group; *p*-values shown in this figure are derived from two-sided unpaired *t*-tests with Welch's correction. **(H)** Western blotting analysis (IB) for RIPK1 and RIPK3 that were immunoprecipitated (IP) with IgG or anti-RIPK1 antibodies.

To explore the potential pathway leading to necrosis in the ISO-treated hearts, we examined the key features of the canonical necroptotic pathway. At 24 h after the second dose of ISO, myocardial protein levels of RIPK1, Ser166-phosphorylated RIPK1 (p-S166-RIPK1), RIPK3, and Ser345-phosphorylated MLKL (p-MLKL) but not total MLKL were significantly increased, whereas myocardial Ser321-phosphorylated RIPK1 (p-S321-RIPK1) significantly decreased, compared with the saline treated group (Figures 2A–G). These changes indicate the activation of the RIPK1–RIPK3–MLKL pathway in the ISO-treated hearts. Another key feature of the activation of the RIPK1–RIPK3 necroptotic pathway is the increased binding of RIPK3 with RIPK1 (44). Our Co-IP experiments revealed a significant increase in RIPK1-bound RIPK3 in ISO-treated hearts than in the saline-treated hearts (Figure 2H).

### Diminishing Isoproterenol-Induced Cardiomyocyte Necrosis by RIPK3 Deficiency in Mice

**Figure 3:**
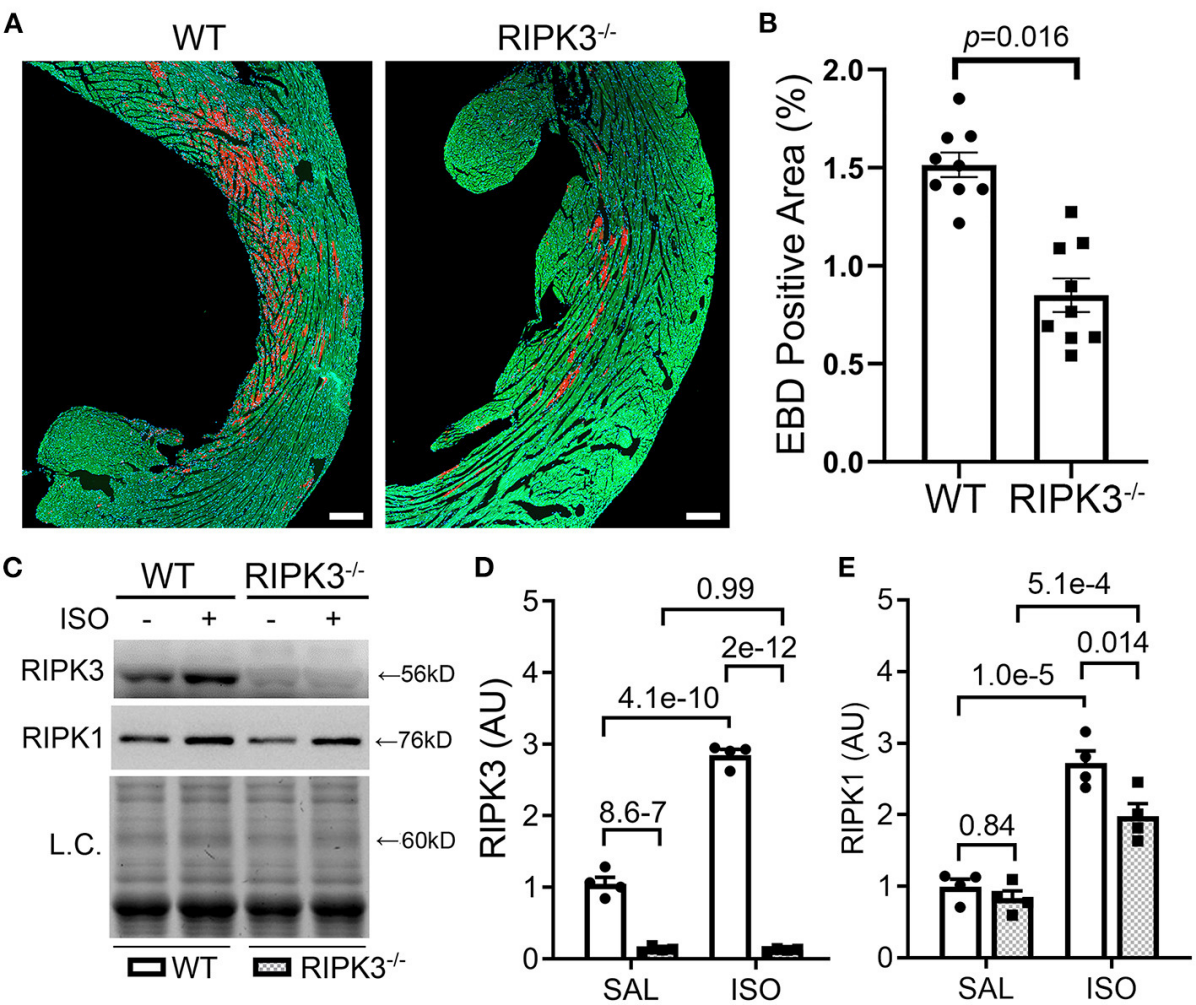
RIPK3 knockout significantly reduced isoproterenol (ISO)-induced cardiomyocyte necrosis. Wild-type and RIPK3 null (RIPK3^−/−^) mice were subjected to the same ISO or saline (SAL) treatment regime and the Evans blue dye (EBD) uptake assays as described in Figure 1. **(A,B)** Representative confocal micrographs **(A)** and pooled morphometric data **(B)** of EBD-positive area. Scale bar = 200 μm. For the EBD assays, three mice (one male and two females) per group and three representative sections per heart were used for morphometry. The *p*-value is shown above the bracket and was obtained from nested *t*-test to account for the three technical repeats within each biological repeat. **(C–E)** Western blotting analyses for myocardial levels of the indicated proteins. Shown are representative image **(C)** and pooled densitometry data **(D,E)**. L.C., loading control that used the stain-free total protein image; mean ± SEM; *n* = 4 mice (two males and two females) per group; two-way ANOVA followed by Tukey's test.

To test whether the ISO-induced cardiomyocyte necrosis belongs to necroptosis and requires RIPK3, we subjected RIPK3^−/−^ and WT mice to the ISO treatment and compared the severity of necrosis between the two groups. EBD assays showed that the same regime of ISO treatment induced ~50% less necrosis in RIPK3^−/−^ mice than in WT mice (Figures 3A,B, *p* = 0.016). Western blotting analyses detected that ISO treatment significantly reduced the increase of myocardial RIPK1 in RIPK3^−/−^ mice than in WT mice (Figures 3C,E). Echocardiograms recorded at 3 h after the second dose of ISO revealed that ISO treatment induced significantly greater increases in left ventricular (LV) ejection fraction (EF) and fractional shortening (FS) and significantly greater decreases in LV chamber diameters at both end-diastole and end-systole in ISO-treated RIPK3^−/−^ mice compared with ISO-treated WT mice (Supplementary Figures II, III), which is well in line with data where the ISO treatment induced significantly less cardiomyocyte loss in RIPK3^−/−^ mice than in WT mice.

### Requirement of RIPK1 Kinase Activity for Isoproterenol to Induce Cardiomyocyte Necrosis

**Figure 4:**
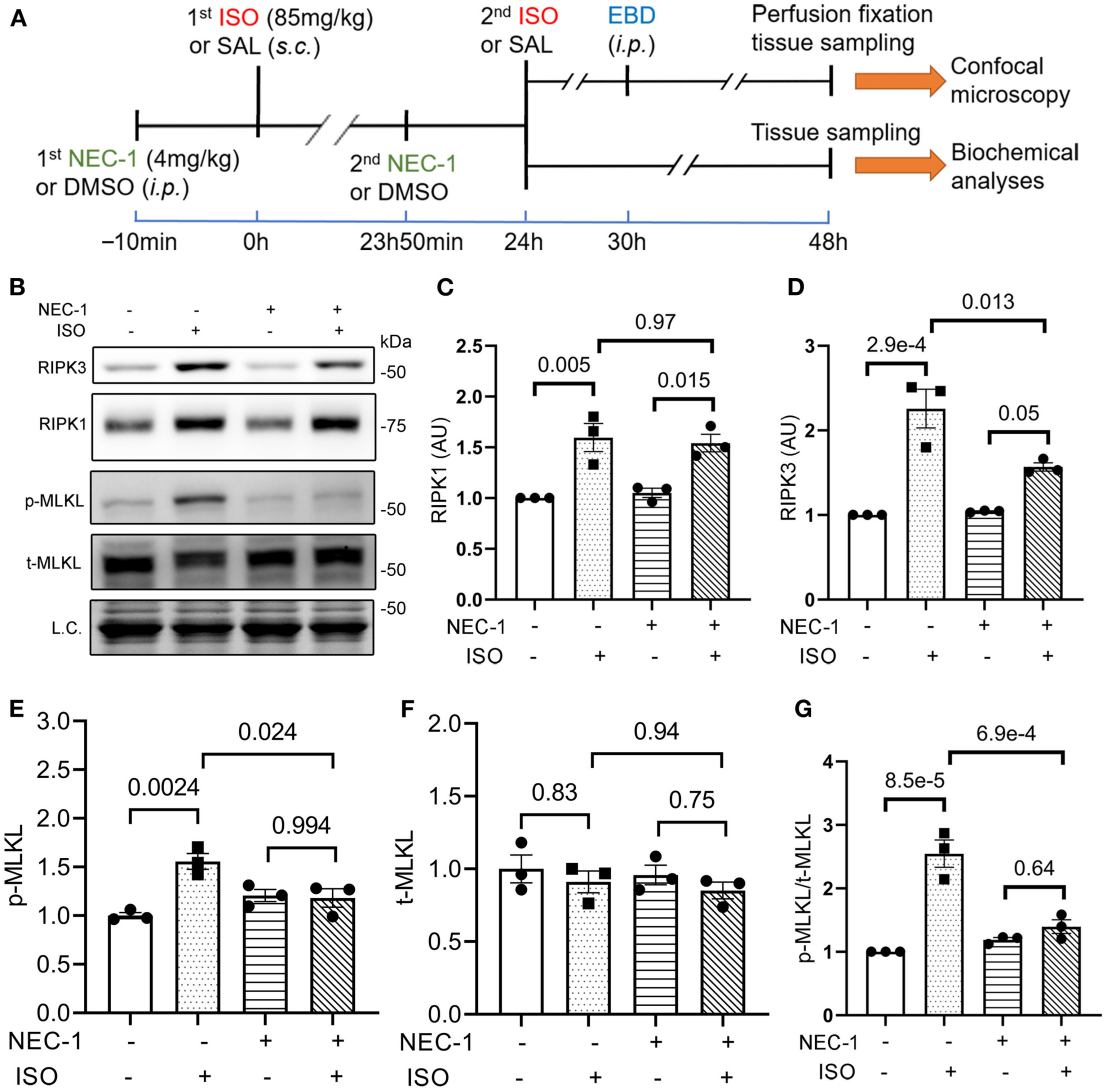
RIPK1 kinase activity is required for the isoproterenol (ISO) treatment to increase RIPK3 and p-MLKL in mouse hearts. **(A)** A schematic for the design of experiments presented in Figures 4, 5. Adult mice were subject to ISO or saline (SAL) treatment as illustrated. Necrostatin-1 (NEC-1; 4 mg/kg, i.p.) or vehicle control (DMSO) was administered 10min before each ISO or SAL injection. **(B–G)** Western blotting analyses for myocardial levels of the indicated proteins. Ventricular myocardium of wild-type (WT) mice was collected 24 h after the second ISO injection. Shown are representative images **(B)** and pooled quantitative data for the indicated proteins **(C–G)**. Two-way ANOVA followed by Tukey's test was used; mean ± SEM; *n* = 3 mice (either two males + one female or one male + two females) per group. L.C. (loading control) used total protein images obtained with the stain-free total protein imaging technology.

**Figure 5:**
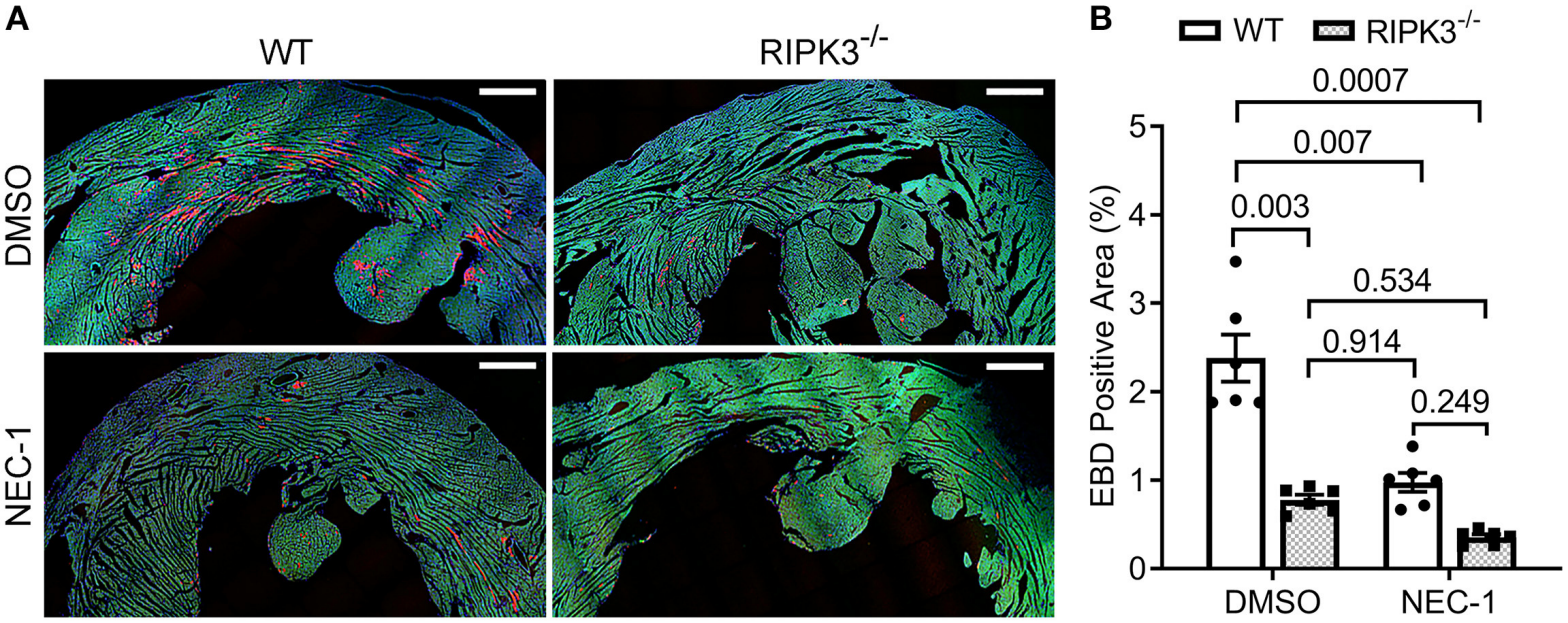
Effect of necrostatin-1 (NEC-1) on isoproterenol (ISO)-induced cardiomyocyte necrosis in wild-type (WT) and RIPK3 knockout mice. WT and RIPK3 null mice were subjected to NEC-1 and ISO treatments as well as the EBD injection as described in Figure 4A. Perfusion-fixed myocardial samples were collected 24 h after the second ISO injection for EBD uptake assays. Shown are representative images (**A**; scale bar = 500 μm) and pooled quantitative data **(B)** from the EBD uptake assays. Two representative sections per mouse and three mice (two males and one female) per group were analyzed; mean ± SEM, nested ANOVA followed by Tukey's test. The nested test is used to account for the two technical repeats (two sections per mouse).

To determine if RIPK1 kinase activity is required for the ISO treatment to induce cardiomyocyte necrosis, we compared the prevalence of ISO-induced cardiomyocyte necrosis in mice pretreated with or without NEC-1, a specific kinase inhibitor of RIPK1 (45). ISO-induced increases in RIPK3 and p-MLKL were remarkably attenuated by the NEC-1 pretreatment, although the protein levels of RIPK1 and total MLKL were not affected (Figure 4). EBD uptake assays revealed that ISO induced significantly less EBD-positive cardiomyocytes in WT mice pre-treated with NEC-1 than in those pretreated with vehicle control. Again, ISO induced significantly less EBD positivity in RIPK3^−/−^ hearts compared with WT mice, but pre-treatment with NEC-1 did not further reduce ISO-induced EBD-positivity in RIPK3^−/−^ mice (Figure 5). These data demonstrate that RIPK1 kinase activity is essential to the induction of cardiomyocyte necrosis by ISO and that RIPK1 and RIPK3 work in the same pathway to mediate ISO-induced cardiomyocyte necroptosis.

## Discussion

Catecholamine surges as well as excessive β-adrenergic stimulation are known to induce cardiomyocyte necrosis, but it was not previously known whether the necrosis or a portion of it belongs to regulated necrosis and, if so, which type it would be. The present study unveils for the first time that a large portion (~50%) of the cardiomyocyte necrosis induced by the ISO regime belongs to necroptosis and is mediated by the RIPK1–RIPK3–MLKL pathway. These are highly significant discoveries because they not only provide a new mechanistic link between many common and rare forms of heart disease and cardiomyocyte necrosis but also identify RIPK1 and RIPK3 as potentially new therapeutic targets for protecting the heart against injury by catecholamine surges or excessive β-adrenergic stimulation.

Cardiomyocyte apoptosis and necrosis are both considered the main modes of cell death in β-adrenergic receptor agonist ISO-induced myocardial injury, but the mechanism underlying necrosis was ill-defined. Here, we confirmed that two consecutive daily doses of ISO (85 mg/kg/day) caused cardiomyocyte necrosis as evidenced by loss of cell membrane integrity in these cells and leukocyte infiltration (Figure 1). More importantly, here, we have established that a large proportion of cardiomyocyte necrosis induced by the ISO treatment belongs to necroptosis because blockade of the canonical necroptotic pathway through either ablation of *RIPK3* gene or inhibition of RIPK1 kinase activity remarkably diminished the ability of the ISO treatment to induce cardiac necrosis in mice. At the same time, our discoveries also demonstrate that the necroptosis induced by ISO is mediated by the RIPK1–RIPK3–MLKL pathway. As elaborated below, these discoveries are compellingly supported by multiple lines of evidence.

First, we have collected strong evidence that the ISO treatment can activate the myocardial RIPK1–RIPK3–MLKL pathway. Autophosphorylation of RIPK1 at serine 166 (p-S166-RIPK1) has been extensively used as a biomarker for RIPK1 activation and was recently shown to be essential for RIPK1 to mediate cell death (both apoptosis and necroptosis) and inflammation (46), whereas phosphorylation of RIPK1 at serine 321 inactivates RIPK1 and thereby prevents TNFα from inducing cell death (47). Our experiments detected that ISO treatment led to significant increases in myocardial p-S166-RIPK1 but marked decreases in p-S321-RIPK1 (Figures 2A–D); hence, the changes in the phosphorylation of both sites reciprocally indicate that RIPK1 is activated in the heart by the ISO treatment. RIPK1 activation can participate in multiple pathways downstream of TNFR1 stimulation; hence, it is important to define which pathway(s) the activated RIPK1 takes in the ISO-treated mice. To this end, our results clearly show that the canonical necroptotic pathway is the main pathway taken by RIPK1 in the ISO treated hearts. This is because (1) myocardial p-MLKL proteins, the most important component of necrosomes in the canonical necroptotic pathway and an indicator of RIPK3 activation (29, 48), were drastically increased in ISO-treated mice (Figures 2A,F); (2) myocardial protein levels of RIPK3, another key component of necrosomes and the central player of the necroptotic pathway (27), were increased by more than 100% (Figures 2A,E); and most importantly, (3) Co-IP revealed that RIPK1-bound RIPK3 was significantly increased in ISO-treated hearts (Figure 2G), a requirement for the participation of RIPK1 in the RIPK3-centered necroptotic activation (44, 49, 50).

Second, we have established that RIPK3 is required for a large proportion of cardiomyocyte necrosis induced by the ISO treatment. This is because the amount of necrosis induced by the same regime of ISO treatment was ~50% less in the RIPK3^−/−^ mice than in WT mice (Figure 3). And RIPK3-dependent necrosis represents an important response of the heart to the ISO treatment as echocardiography showed that the ISO-treatment induced greater increases in EF and FS and greater concentric hypertrophy in RIPK^−/−^ mice than in WT mice (Supplementary Figures II, III).

Lastly, our experiments showed that RIPK1-selective inhibition with NEC-1 did not discernibly affect the RIPK1 protein levels but significantly attenuated RIPK3 protein increases and nearly completely blocked the upregulation of p-MLKL in the ISO-treated mice (Figure 4). Consistent with the biochemical changes, EBD uptake assays revealed that NEC-1 pre-treatment reduced ISO-induced EBD positivity by ~60% in WT mice and RIPK3 deficiency yielded a similar effect, but NEC-1 failed to further reduce necrosis in RIPK3-deficient mice (Figure 5). These new experimental findings demonstrate that RIPK1 kinase activity is required for, and RIPK1 and RIPK3 work in the same pathway in, mediating ISO-induced necroptosis.

Notably, crosstalk among different cell death pathways has been documented. Activation of death receptors usually induces apoptosis *via* the extrinsic pathway, but inhibition of caspase 8 switches it to the canonical necroptotic pathway (RIPK1–RIPK3–MLKL) (22). Oligomerization of p-MLKL at the cell membrane serves as the executioner of necroptosis, which differentiates necroptosis from other types of lytic cell death (51). During innate immune responses, program cell death also can be in another form of regulated necrosis, pyroptosis (52). In pyroptosis, inflammasomes are formed in response to pathogen-associated and damage-associated molecular patterns (PAMPs and DAMPs, respectively), leading to the recruitment of apoptosis-associated speck-like protein containing a CARD (ASC), followed by the recruitment and self-activation of caspase-1; activated caspase 1 processes other molecules including the executioner of pyroptosis, gasdermin D (GSDMD). Similar to the function of p-MLKL in necroptosis, the N-terminal fragment of GSDMD resulting from the caspase 1-mediated proteolytic cleavage undergoes oligomerization to form pores within the cell membrane and thereby cause the cell to swell and burst (52). More recently, the collective activation of all the three programmed cell death pathways (apoptosis, pyroptosis, and necroptosis) in the same population of cultured cells by microbial infection was observed and termed “PANoptosis” (51). The concept of PANoptosis as a form of inflammatory cell death is still in its infancy. Some molecules such as Z-DNA-binding protein (ZBP1) and transforming growth factor β-activated kinase 1 (TAK1) were found to regulate all the three forms of programmed cell death covered by PANoptosis. ZBP1 seemed to be crucial for the activation of all the three pathways by influenza A virus (IAV) infection (53, 54). Inhibition of TAK1 activity by genetic deletion or pathogen-mediated inhibition also activates pyroptosis, apoptosis, and necroptosis (55–58). Physical interactions between molecules known to participate in apoptosis, pyroptosis, and necroptosis have been reported in the cell death induced by the loss of TAK1 (58). A cell death complex composed of key molecules from pyroptosis, extrinsic apoptosis, and necroptosis in the activation of PANoptosis in cultured cells by certain types of bacterial and viral infections was detected by immunoprecipitation and termed the PANoptosome (59). Therefore, there is a possibility that the activation of RIPK1 and RIPK3 and the formation of the RIPK1–RIPK3 complex in the ISO-treated myocardium might be a part of PANoptosis rather than independent necroptosis. However, as evidenced by the marked increases of p-MLKL in ISO-treated hearts (Figure 2) and the nearly complete abolishment of the increase in p-MLKL by RIPK1 kinase inhibition (Figure 4), increased p-MLKL is intimately involved in the regulated necrosis induced by the ISO treatment. Thus, it is more than likely that necroptosis is a major form of regulated necrosis induced by ISO. It will be very interesting to test in the future whether pyroptosis and PANoptosis are also involved.

Although β-adrenergic activation *per se* should exert vasodilation effects, coronary insufficiency and even myocardial ischemia were observed in the same ISO-induced myocardial injury model as the one used in the present study (21). In fact, this model has been used by many as a non-invasive MI model (21). It is conceivable that myocardial I/R occur in this model; and the cardiac histopathology in this model does resemble MI and I/R injury (21). However, the massive cardiomyocyte necroptosis in this model makes it different from a surgically induced MI model. This is because a prior report shows quite convincingly that RIPK3-mediated necroptosis is not a discernible contributor to the acute infarct size in a mouse model of acute MI induced by coronary artery ligation, although RIPK3 deficiency did attenuate the chronic post-MI maladaptive cardiac remodeling (32). The requirement of RIPK1 kinase activity and the apparent involvement of MLKL in mediating the ISO-induced necroptosis also seem to distinguish this model from a traditional myocardial I/R injury model. This is because a recent high-profile study by Zhang et al. showed that cardiac necroptosis triggered by I/R injury required RIPK3 but not RIPK1 and MLKL. They showed that RIPK3 upregulated by I/R phosphorylates and activates the calcium/calmodulin-dependent protein kinase II (CaMKII) and thereby opened the MPT to induce cardiomyocyte necroptosis (33). However, more recent studies suggest that the RIPK3–MLKL axis may still be important for myocardial necroptosis in I/R injury (34). Moreover, the canonical RIPK1–RIPK3–MLKL pathway has been implicated in cardiomyocyte necroptosis induced by genetic interrogations of key cellular processes in mice; the perturbation of Cullin deneddylation by cardiomyocyte-restricted ablation of *Cops8* gene and the suppression of nuclear DNA-encoded mitochondrial genes required for ATP synthesis due to the knockout of the Hippo signaling effector TEAD1 are among the examples (41, 60, 61). It is well-known that induction of necroptosis by the activation of TNFR1 requires RIPK1 kinase activity (26). Qin et al. reported that myocardial I/R could dysregulate both strands (5p and 3p) of miR-223 in mice, thereby targeting TNFR1 and other regulatory points upstream of RIPK3 to cause cardiac necroptosis (62). By definition, necroptosis and the necrosis driven by the MPT opening are two different types of regulated necrosis (22). Necroptosis can be induced in cells without mitochondria (63). Hence, it will be interesting and important to test in future studies whether CaMKII plays a mediating role in ISO-induced cardiomyocyte necrosis, as CaMKII can be activated by excessive β-adrenergic stimulation.

**Figure 6:**
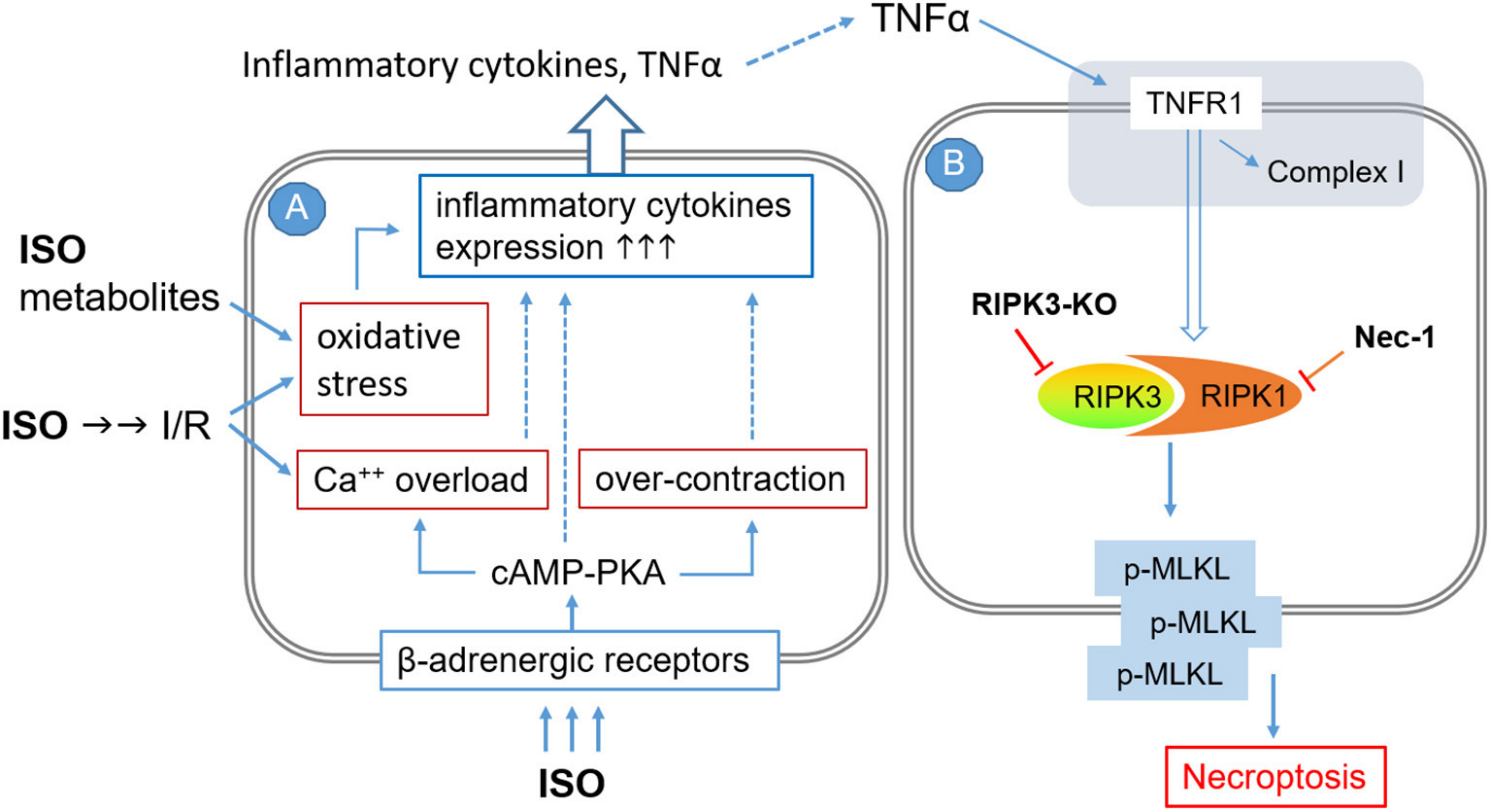
A working model for the potential mechanisms by which high doses of isoproterenol (ISO) induce cardiomyocyte necroptosis. The pathological processes are illustrated to occur sequentially in two adjacent cardiomyocytes (**A** and **B**), but they could potentially take place in the same cell where TNFα and other inflammatory cytokines would be autocrinal. Processes shown in cell **A** are based on previous reports. The pathway modeled in cell **B** is based on the findings of the present study, but the involvement of TNFα and its receptor (shaded area at the top) as well as the requirement of MLKL for the induction of cardiomyocyte necrosis by ISO remain to be fully established. I/R, ischemia/reperfusion; TNFR1, TNFα receptor type 1; RIPK3-KO, RIPK3 knockout; NEC-1, necrostatin-1.

ISO infusion at a low dose (e.g., 12.5 μg/kg/h) that is capable of inducing cardiac hypertrophy but not fibrosis was shown to rapidly upregulate the gene expression of TNF and other inflammatory cytokines including interleukin (IL)-1β, IL-6, inducible nitric oxide synthase, and monocyte chemotactic protein-1 (MCP-1) in a TNFR1-dependent manner (64). Myocardial TNFα and IL1β protein levels were markedly increased in rats treated with two consecutive daily doses of ISO (100 mg/kg/day, i.p.) (37). Hence, it is very likely that autocrinal or paracrinal TNFα and its activation of TNFR1 mediate the activation of the RIPK1–RIPK3–MLKL pathway by the ISO treatment (Figure 6), although no PubMed searchable studies have tested the requirement of TNFR1 in the induction of cardiomyocyte necrosis by catecholamine surges yet. As illustrated in Figure 6, several previously reported pathological processes also could serve as the upstream events for the inflammatory responses and cardiac injury. These include, for example, increased oxidative stress resulting from myocardial I/R and catecholamine metabolism, calcium overload and myofibril over-contraction as a result of excessive β-adrenergic receptor activation and coronary insufficiency, and instant cardiomyocyte necrosis caused by myocardial ischemia and other factors (19, 21, 36).

Since pharmacological inhibition of RIPK1 and genetic ablation of RIPK3 prevented a large proportion of ISO-induced cardiomyocyte necrosis, the present study provides a strong argument for targeting RIPK1 or RIPK3 to protect against cardiac injury from catecholamine surges and against maladaptive cardiac remodeling induced by excessive β-adrenergic activation. Given that catecholamine surges play an important role in a broad spectrum of diseases, including stress cardiomyopathy that has been intimately associated with the physical and emotional stress resulting from the current COVID-19 pandemic (10–13), the present study provides a serendipitous and yet strong support for targeting RIPK1 and RIPK3 to treat COVID-19. This is actually very exciting and plausible because both RIPK1 and RIPK3 play critical roles in inflammation (65, 66). And at least two RIPK1 inhibitors have passed through Phase I clinical trials, and many chemical inhibitors of RIPK3 are emerging (67, 68). In fact, several recent reports suggested exploring RIPK1 and RIPK3 as drug targets for COVID-19 (69–71).

### Limitation of the Study

The bulk of the experiments of this study was carried out during the COVID-19 pandemic; hence, the experimental design was streamlined. For example, the number of animals per group could have been greater, and the readouts for cardiac injury such as the leakage of cardiac enzymes to the circulation and the effects of RIPK-KO and NEC-1 treatment on myocardial inflammatory responses (e.g., leukocyte infiltration) and other forms of regulated cell death (e.g., apoptosis and pyroptosis) also could have been determined more extensively along with necroptosis, to get a more complete picture. Nonetheless, we contend that the evidence presented here compellingly supports the main conclusions that unveil a molecular pathway that mediates cardiomyocyte necrosis induced by catecholamine surges, a timely and mechanistic discovery that probably identifies new therapeutic targets for treating cardiac injury induced by catecholamine surges.

## Data Availability Statement

The original contributions presented in the study are included in the article/Supplementary Material, further inquiries can be directed to the corresponding author/s.

## Ethics Statement

The animal study was reviewed and approved by The Institutional Animal Care and Use Committee (IACUC) of the University of South Dakota.

## Author Contributions

XW, PW, and JL: conception and experimental design. PW and MC: data collection and interpretation. PW and XW: manuscript preparation. All authors contributed to the article and approved the submitted version.

## Funding

This study was in part supported by NIH grants HL072166, HL085629, HL131677, and HL153614 (to XW).

## Conflict of Interest

The authors declare that the research was conducted in the absence of any commercial or financial relationships that could be construed as a potential conflict of interest.

## Publisher's Note

All claims expressed in this article are solely those of the authors and do not necessarily represent those of their affiliated organizations, or those of the publisher, the editors and the reviewers. Any product that may be evaluated in this article, or claim that may be made by its manufacturer, is not guaranteed or endorsed by the publisher.
