# Supplementary material for: Catecholamine Surges Cause Cardiomyocyte Necroptosis via a RIPK1–RIPK3-Dependent Pathway in Mice
Source: Front Cardiovasc Med. 2021 Sep 16;8:740839. doi: 10.3389/fcvm.2021.740839 (PMC8481609; doi:10.3389/fcvm.2021.740839)
Supplement: Supplementary file 1 [file Data_Sheet_1.PDF]

Supplementary Data to

Catecholamine Surges Cause Cardiomyocyte Necroptosis via a RIPK1-RIPK3 Pathway in Mice

Penglong Wu[1,2], MD, PhD; Mingqi Cai[1], MD; Jinbao Liu[2], MD, PhD; Xuejun Wang[1]*, MD, PhD

I. Supplementary Methods

II. Supplementary Figures I ~ III

I. Supplementary Methods

Echocardiography

Trans-thoracic echocardiography was performed on mice using the VisualSonics Vevo 2100 system and a 30 MHz probe as previously described.(Pan et al., 2020) Mice were kept in light anesthesia with inhalation of 1.5% isoflurane in room air supplemented with 100% oxygen when echocardiography was recorded. A 2D-mode short axis view was recorded at the level of papillary muscle and 2D-guilded M-Mode echocardiograms acquired through the anterior and posterior walls. Primary measurements of the end-diastolic and the end-systolic left ventricular (LV) posterior wall thickness and LV end-diastolic and end-systolic chamber dimensions were used to derive functional parameters, such as fractional shortening (FS) and ejection fraction (EF).

II. Supplementary Figures

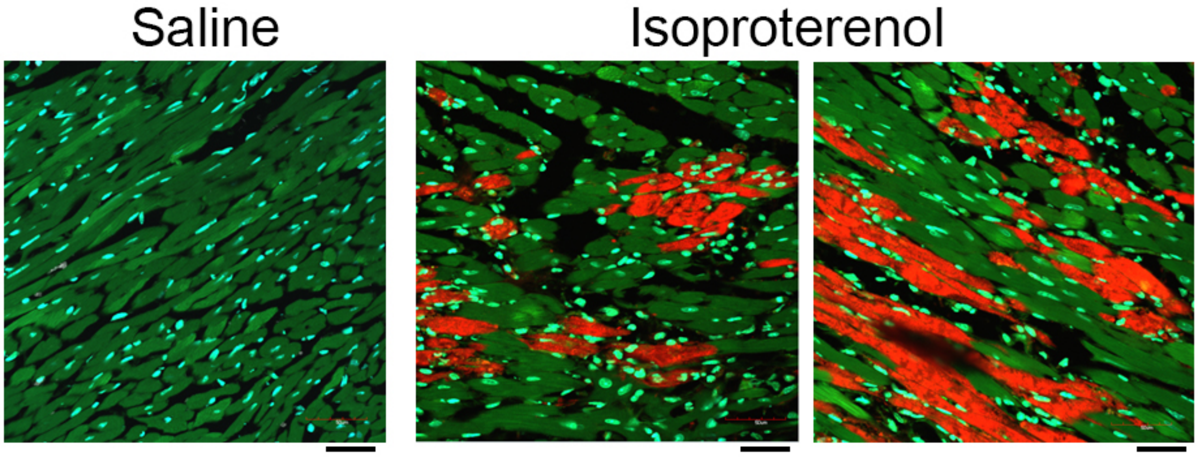

Supplementary Figure I. Higher magnification of confocal micrographs of left ventricular myocardium from mice at 24 hours after the second daily injection of isoproterenol or vehicle control (saline). Mice were treated and the tissue samples were processed and stained as described in Figure 1A of the main text. Scale bar = 30µm.

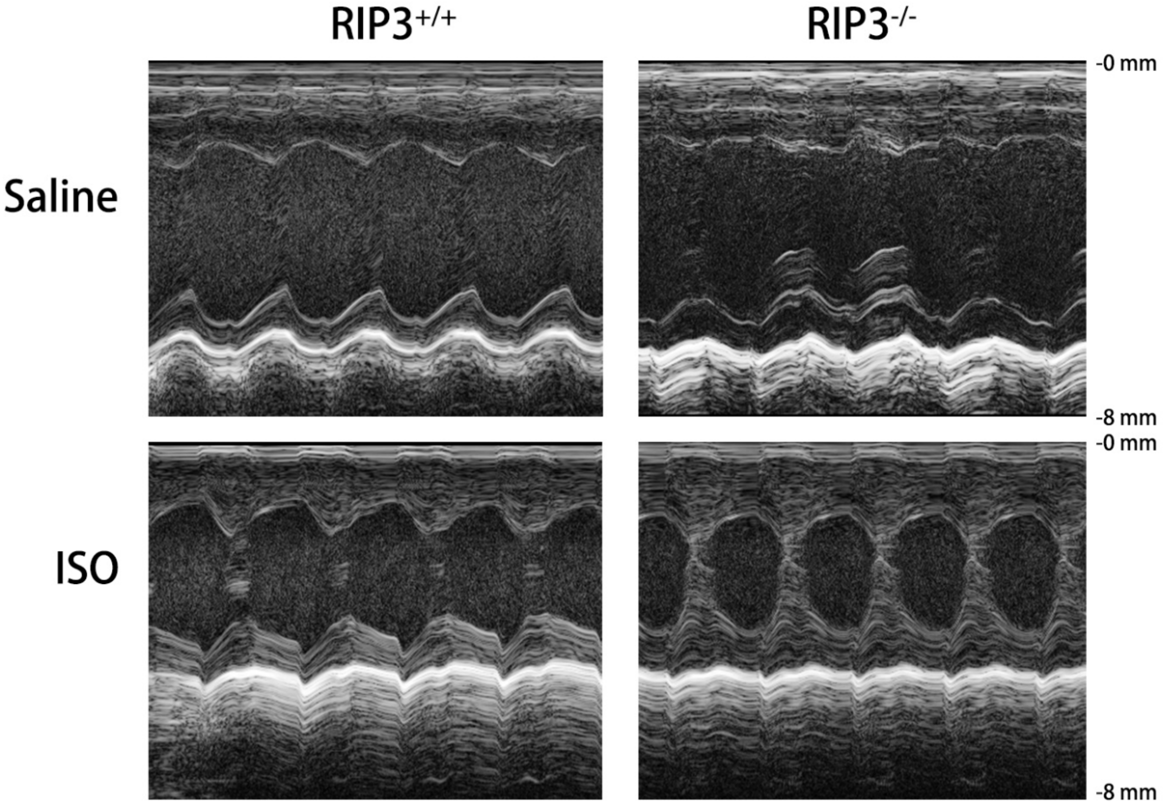

Supplementary Figure II. Representative M-mode echocardiograms. WT and RIPK3 null mice were subjected to 2 consecutive daily injections of ISO or saline (SAL) as described in **Figure 4A** of the main text. Echocardiography was performed 3 h after the second injection.

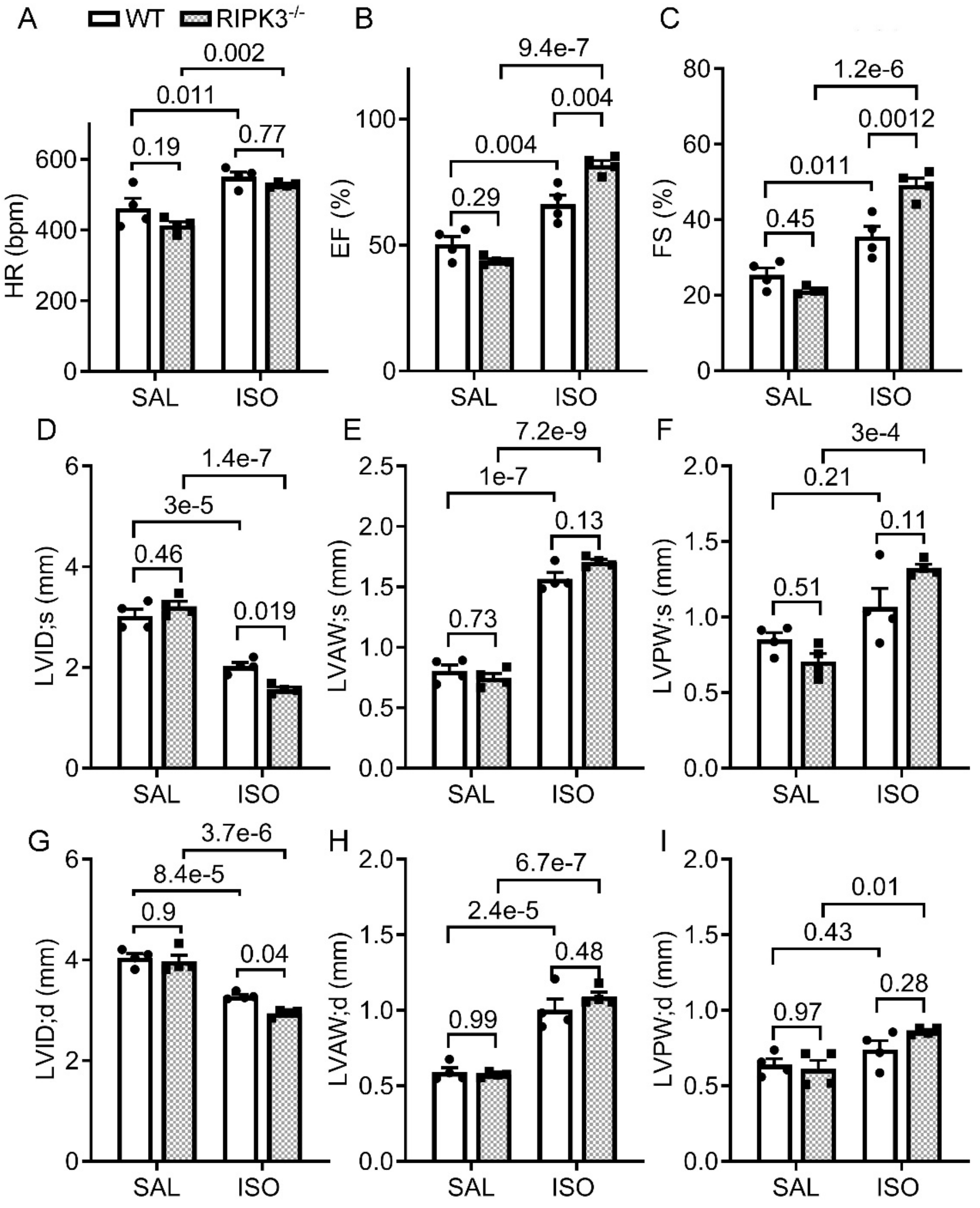

Supplementary Figure III. Impact of RIPK3 deficiency on isoproterenol (ISO)-induced mouse LV morphometric changes. WT and RIPK3 null mice were subjected to 2 consecutive daily injections of ISO or saline (SAL) as described in **Figure 4A** of the main text. Echocardiograms were recorded 3 h after the second injection. HR, heart rate; bpm, beats per minute; EF, ejection fraction; FS, fraction shortening; LV, left ventricle; LVAW;s/d: end-systolic/diastolic LV anterior wall thickness; LVPW;s/d, end-systolic/diastolic LV posterior wall thickness; LVID;s/d, end-systolic/diastolic LV internal dimension; mean±SEM; n=4 mice per group; two-way ANOVA followed by Tukey's test was used.
